# Supplementary material for: Engineered spin-orbit interactions in LaAlO3/SrTiO3-based 1D serpentine electron waveguides
Source: Sci Adv. 2020 Nov 25;6(48):eaba6337. doi: 10.1126/sciadv.aba6337 (PMC7688326; doi:10.1126/sciadv.aba6337)
Supplement: http://advances.sciencemag.org/cgi/content/full/6/48/eaba6337/DC1 [file supp_6_48_eaba6337__index.html]

Science Advances | Science AdvancesAAASSearchScience AdvancesMenu

## Supplementary Materials

# Engineered spin-orbit interactions in LaAlO3/SrTiO3-based 1D serpentine electron waveguides

Megan Briggeman, Jianan Li, Mengchen Huang, Hyungwoo Lee, Jung-Woo Lee, Kitae Eom, Chang-Beom Eom, Patrick Irvin, Jeremy Levy

Download Supplement

**This PDF file includes:**

- Figs. S1 and S2

**Files in this Data Supplement:**

- Adobe PDF - aba6337\_SM.pdf
