# Supplementary material for: Engineered spin-orbit interactions in LaAlO3/SrTiO3-based 1D serpentine electron waveguides
Source: Sci Adv. 2020 Nov 25;6(48):eaba6337. doi: 10.1126/sciadv.aba6337 (PMC7688326; doi:10.1126/sciadv.aba6337)
Supplement: http://advances.sciencemag.org/cgi/content/full/6/48/eaba6337/DC1 [file supp_6_48_eaba6337__1.pdf]

Science Advances
AAAS

advances.sciencemag.org/cgi/content/full/6/48/eaba6337/DC1

Supplementary Materials for

Engineered spin-orbit interactions in $LaAlO_3/SrTiO_3$-based 1D serpentine electron waveguides

Megan Briggeman, Jianan Li, Mengchen Huang, Hyungwoo Lee, Jung-Woo Lee, Kitae Eom, Chang-Beom Eom, Patrick Irvin, Jeremy Levy*

*Corresponding author. Email: jlevy@pitt.edu

Published 25 November 2020, *Sci. Adv.* **6**, eaba6337 (2020)
DOI: 10.1126/sciadv.aba6337

This PDF file includes:

Figs. S1 and S2

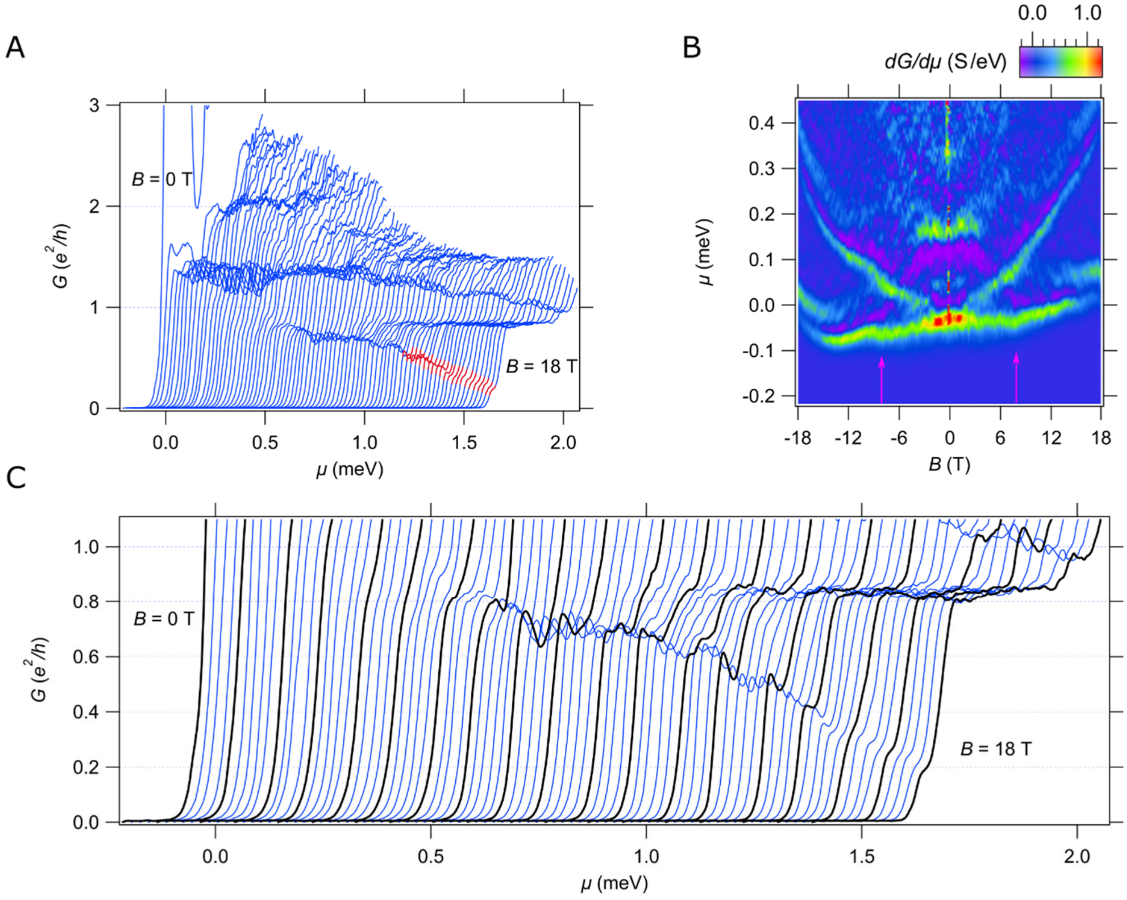

Figure S1. Magnetotransport for serpentine superlattice Device B. (A) Conductance G as a function of chemical potential μ for Device B. Curves are at different applied out-of-plane magnetic field values from 0 T to 18 T. A fractional conductance feature is observed below the $\sim e^2/h$ plateau at high magnetic fields (highlighted in red). **(B)** Transconductance map as a function of magnetic field B and chemcial potential μ. An overall drift while gating effects the subband structure of this device. **(C)** Zoom-in of the high-field fractional conductance feature.

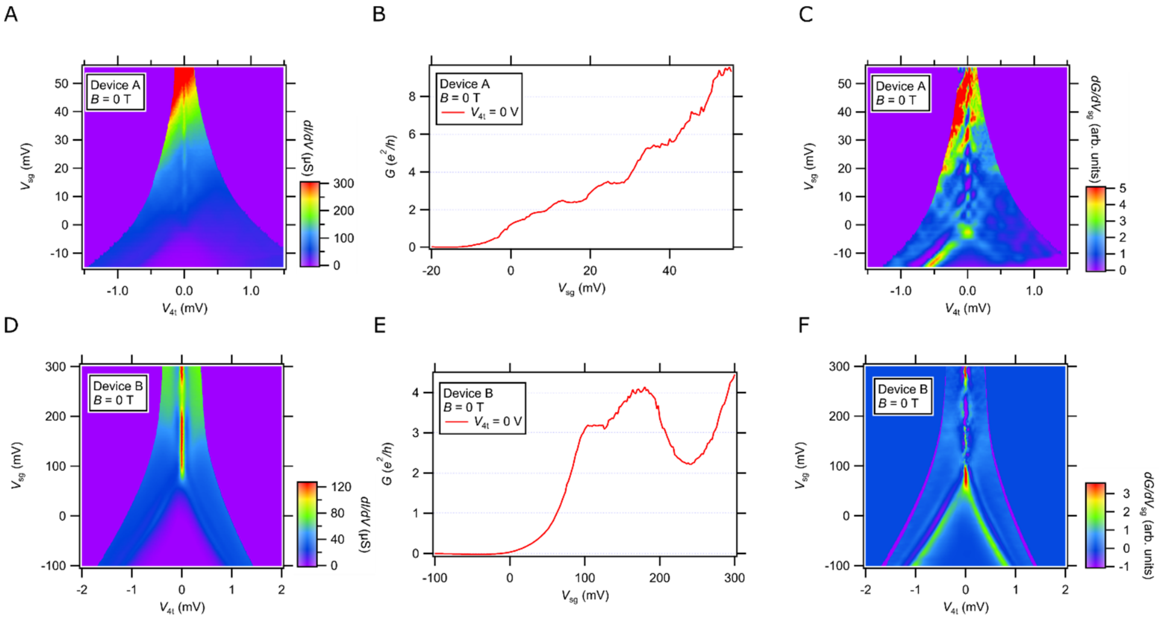

Figure S2. Finite-bias spectroscopy for Devices A and B. (A) Conductance dI/dV as a function of four-terminal voltage V_{4t} and side gate voltage V_{sg} for Device A at $B = 0$ T. **(B)** Zero bias ($V_{4t} = 0$ V) conductance line cut of Device A. **(C)** Transconductance map dG/dV_{sg} as a function of four-terminal voltage and side gate voltage. **(D)** Conductance dI/dV for Device B. This device shows signatures of superconductivity near zero bias. **(E)** Conductance linecut for Device B. **(F)** Transconductance dG/dV_{sg} for Device B. All data taken at $B = 0$ T.
